# Biocompatible Electrical and Optical Interfaces for Implantable Sensors and Devices

**DOI:** 10.3390/s24123799

**Published:** 2024-06-12

**Authors:** Yuxin Wan, Caiyi Wang, Bingao Zhang, Yixuan Liu, Hailong Yang, Fengyu Liu, Jingjing Xu, Shengyong Xu

**Affiliations:** 1 Key Laboratory for the Physics and Chemistry of Nanodevices, School of Electronics, Peking University, Beijing 100871, China; 2 School of Integrated Circuits, Shandong University, Jinan 250100, Chinaxujj@sdu.edu.cn (J.X.); 3 Key Laboratory for Neuroscience, Neuroscience Research Institute, Department of Neurobiology, School of Basic Medical Sciences, Ministry of Education and National Health Commission, Peking University, Beijing 100191, Chinaliufyu@bjmu.edu.cn (F.L.)

**Keywords:** implanted sensor, electrical interface, optical interface, biocompatibility, human–machine integration

## Abstract

Implantable bioelectronics hold tremendous potential in the field of healthcare, yet the performance of these systems heavily relies on the interfaces between artificial machines and living tissues. In this paper, we discuss the recent developments of tethered interfaces, as well as those of non-tethered interfaces. Among them, systems that study neural activity receive significant attention due to their innovative developments and high relevance in contemporary research, but other functional types of interface systems are also explored to provide a comprehensive overview of the field. We also analyze the key considerations, including perforation site selection, fixing strategies, long-term retention, and wireless communication, highlighting the challenges and opportunities with stable, effective, and biocompatible interfaces. Furthermore, we propose a primitive model of biocompatible electrical and optical interfaces for implantable systems, which simultaneously possesses biocompatibility, stability, and convenience. Finally, we point out the future directions of interfacing strategies.

## 1. Introduction

In an era of rapidly advancing biotechnology and electronics, the integration of humans with machines has become a hot topic in the research community, showing immense application potential in interdisciplinary fields such as neuroscience, rehabilitation medicine, and bioengineering [1,2,3,4,5,6]. The current instances of human–machine integration can be divided into two main categories: invasive systems and noninvasive systems. The former require relatively complicated surgical procedures to implant sensing or actuating modules into the human body, increasing the risk of infection. The latter, however, utilize external devices, involving wearable devices to realize the human–machine interaction, at the expense of an inferior signal quality.

During this trend, implantable sensors and devices have undergone unprecedented revolutionary breakthroughs, offering promising applications across diverse domains, from diagnostics to therapeutics. In terms of medical assessment, there are numerous methods using electrodes and probes to detect and analyze physiological signals, including electrocorticography (ECoG) [7,8], micro-electrocorticography (μECoG) [9,10], electroencephalography (EEG) [11,12], electromyography (EMG) [13,14], and others [15,16,17,18,19], which provides an important means to gain a deeper understanding of brain and muscle activity. Similarly, to treat diverse functional diseases, a great many implantable systems have been approved, such as cardiac pacemakers for cardiac dysfunction and cochlear implants for hearing impairment, significantly improving the quality of life of patients and bringing new possibilities to the medical field [20,21,22].

Establishing stable, effective, and biocompatible interfaces between artificial machines and living tissues plays a vital role in implantable bioelectronics since the interface technologies inevitably determine the transmission of experimental signals, as well as the responses of surgical wounds. Hereinafter, the term “electrical interface” refers to an electrical feedthrough mounted onto a piece of skin or bone, usually with multiple channels connecting the artificial sensors or devices implanted into live bodies to external instruments so that electrical signals can be read out or sent in. The term “optical interface” refers to an optical feedthrough which allows light to pass through freely from either side. The following reviews the different kinds of interfacing modules in implantable systems, encompassing physical tethers and other specially shaped interfaces. We also discuss the selection of the perforation site, the fixing strategy for interfaces, the precautions with long-term indwelling, and the properties of wireless systems as a comparison. Moreover, we present a versatile model of a multi-functional interface that is biologically friendly and conveniently usable.

## 2. Tethered Interfaces

Connecting devices in vitro and/or in vivo in laboratory versions of implantable systems commonly relies on physical tethers that are convenient to operate and available for purchase. For instance, copper wires are frequently used in neuroscience research, whereby anisotropic conductive film (ACF) or silver paste one end of the wire is adhered to an electrode for measurement or stimulation and the other to an external instrument for data processing or signal controlling. Likewise, commercial optical fibers and fluidic tubes serve as an interfacing module in optogenetic experiments and drug delivery, respectively.

### 2.1. Multi-Channel Interfaces

The properties of the physical tethers determine the signal quality and quantity to a large degree, so using a single commercial cable may not be the optimal interface strategy for implantable systems in more complicated contexts.

**Figure 1:**
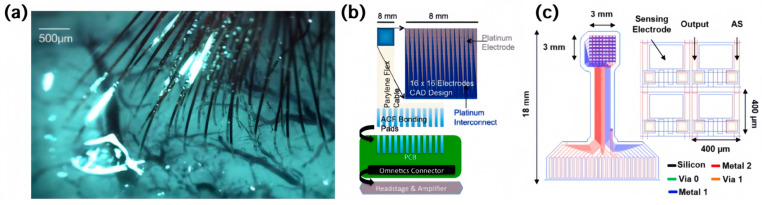
Different layouts of cables in multi-channel interfaces. (**a**) Arrays of electrode threads inserted into the cerebral cortex [23]; (**b**) schematic drawing of a 256-channel μECoG measurement system [24]; (**c**) schematic drawing of a double-layer metal interconnected cable [25].

One trend in interface research is scaling the number of channels to meet the pressing need for increased signal density. As is depicted in Figure 1a, a customized neurosurgical robot is built to rapidly and reliably insert arrays of 96 electrode threads. The arrays are packaged into a small device equipped with other sensing and processing modules, making electrophysiological recordings over 3000 channels. In addition to insertion on the surface of the cerebral cortex, the system can also be positioned and implanted into multiple brain regions to achieve a broader range of neural signal recording. The main substrate and medium used in this ultra-thin polymer probe is polyimide (PI), whose dimensions and composition match the material properties of brain tissue, allowing for long-term implantation in living organisms [23]. But the above interfacing strategy is not generally applicable. The individual cabling of each channel is undesirable in that it requires opening wounds across large areas to arrange the wires in a distributive pattern or extremely delicate surgical techniques for small-interval arrays. Researchers are thus exploring ways to fabricate multi-channel cables. The mainstream process is metal deposition and etching on thin film substrates. In one example, platinum is e-beam-evaporated and patterned in parylene using oxygen plasma reactive ion etching (Figure 1b). This cable is monolithically integrated with the electrode array at the wafer level and thermo-compression-bonded to a fanout plastic circuit board (PCB) in an ACF flex process, forming a 256-channel μECoG measurement system. The integration of the cable with the electrode array allows for precise positioning of the electrodes in the target brain regions, enabling accurate and localized neural recordings. The thermo-compression bonding process ensures a secure and reliable connection between the parylene cable and the fanout PCB, enhancing the overall stability and performance of the μECoG system. The use of ACF technology facilitates efficient signal transmission and data acquisition across all 256 channels [24]. Figure 1c shows an improved structure: a double-layer metal interconnected cable that realizes a great rise in the channel count to the kilo-scale and mega-scale. During the implantation process, the interconnected cables outside the electrode array are bent at a 90° angle with a bending radius of less than 2 mm to reduce the formation of tiny cracks during cable handling. Furthermore, to enhance the stability of the interconnected cable region, a 12.5 μm thick PI protective layer is added to the top surface of the array cables. It is notable that in this work, thermally grown silicon dioxide serves as a robust, bendable, and ultrathin biofluid barrier [25,26].

Recently, some groups have adopted new methods to establish multi-channel interfaces, involving 3D printing [27], liquid metal injection [28], and thermal drawing processes [29], but these methods are still in an embryonic stage, where one cable contains tens of channels and specialized cables are only feasible for set experimental objectives.

### 2.2. Multi-Functional Interfaces

Other interfaces of recent interest are concerned with integrating multiple functions, such as electrical stimulation and photometric recording [30,31,32]. Through innovation in the constructional design and manufacturing processes, a minimally invasive interface is able to transmit two or more separate signals simultaneously.

**Figure 2:**
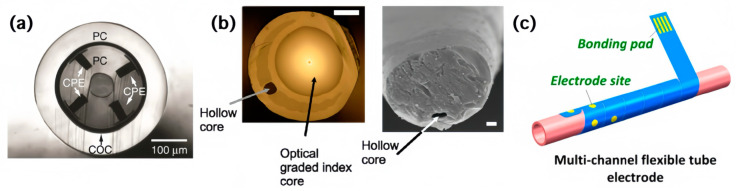
Different geometric designs of tethers in multi-functional interfaces. (**a**) Cross-sectional optical image of a fiber probe integrating one microfluidic channel, four electrodes, and a surrounding waveguide [29]; (**b**) photomicrograph of the dual-core fiber (left) and Scanning Electron Microscope micrograph of the tip (right) [33]; (**c**) schematic drawing of the flexible tube with electrodes distributed on the surface [34].

The concentric preform, as depicted in Figure 2a, consists of a central hollow microfluidic channel surrounded by a polycarbonate (PC) cylindrical waveguide and four conductive polyethylene (CPE) electrodes. The preform is subjected to the thermal drawing process, resulting in a micro-diameter fiber, with its dimensions reduced by a factor of approximately 100. To further reduce its dimensions, dichloromethane is used to selectively etch the outer PC cladding, exposing the inner components of the fiber. These multifunctional fiber probes have been implanted into the medial prefrontal cortex (mPFC) of mice. They interact with the external world by connecting to external circuit boards and optical fibers, realizing optogenetic stimulation, neural recording, and drug delivery. These probes form stable brain–machine interfaces in the mouse brain, allowing for long-term implantation and functional maintenance and facilitating extended manipulation and analysis of the neural circuits [29].

Figure 2b demonstrates a different geometry of the fiber instead of a concentric structure. It contains two cores in a silica inclusion tube: a graded refractive index optical core doped with GeO_2_ for light delivery and collection and a silica capillary hole filled with an electrolyte solution (1–3 M NaCl) to record electrical activity. The tapered end of the optical fiber probe is typically implanted into the intact brain tissue of a mouse, while the other end is connected to a photomultiplier tube (PMT) via a multimode optical fiber, undertaking the transmission, collection, and electrical recording of light [33]. Along with these cylindrical fibers having been inspired by the stack-and-draw method, exploiting their surface structure to develop a multi-functional interface has also been validated. For example (Figure 2c), wrapping a sandwich-structured parylene electrode onto the surface of a PI capillary enables omnidirectional electrical interaction with tissue, as well as oriented nutrition factor delivery. The microelectrode structure is composed of a hollow tubular core acting as a fluidic channel and electrode sites distributed on the surface of the tubular structure. This design allows for contact with tissue from any direction. The microelectrode has been implanted into the gastrocnemius (GA) and tibialis anterior (TA) muscles of a rat, implementing dynamic electrophysiological recording and fluidic drug delivery. The electrical stimulation sequence and electrophysiological signals could be imported and extracted through the electrode sites, facilitating bidirectional communication with the surrounding tissue [34].

## 3. Non-Tethered Interfaces

Tethered interfaces concentrate mainly on signal transmission but lack consideration of the tissues around. In order to establish better contact that is mechanically secure and biologically friendly, interfaces that are untethered have emerged in recent years. One natural and popular design is to substitute artificial electrical systems for artificial human body parts, in which case their biomimetic geometry distributes the mechanical stress around the interface and inhibits the impact of tissue regeneration. For those whose target organs are far from the surface of the body, additional thought should be given to the inner channel and the percutaneous structure.

### 3.1. Artificial Dura Systems

An artificial dura system is one of the broadly employed interfaces for implants that have direct contact with the brain or spinal cord. It can be traced to cranioplasty, a centuries-old surgical procedure for cranial defect repair [35]. Inspired by bone grafts for cranioplasty, artificial dura systems utilize metals such as titanium to replace the skull, supporting the inner devices and protecting the brain. To satisfy the demand of optical imaging, the past two decades have witnessed the development of a transparent artificial dura system, which typically constitutes dural substitute and functional modules, with glass cranial windows positioned over the top of the implanted devices [36,37,38].

**Figure 3:**
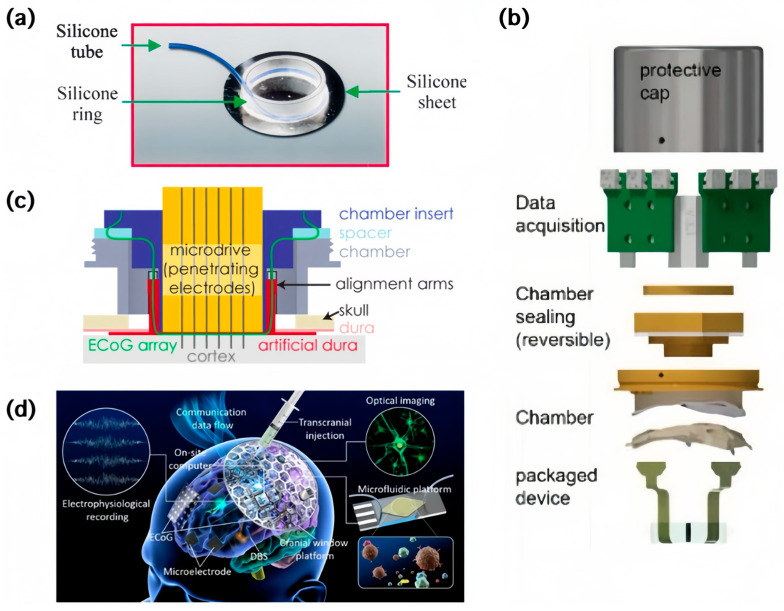
Examples of artificial dura systems. (**a**) A silicone dura substitute for long-term optical imaging [39]; (**b**) schematic drawing of a chamber-based system for neural activity [25]; (**c**) schematic drawing of a chamber system that simultaneously records μECoG, LFP, and spiking activity [40]; (**d**) a proposal for large-area multi-functional artificial dura system [41].

Figure 3a illustrates an early silicone design consisting of a sheet to cover the cortical surface, a ring to block the re-growth of the cutting edge of the natural dura, and a tube to let different solutions be flushed over the cortex. A glass coverslip serves as a cranial window, and the whole device is shielded by a metal chamber and a metal cap. This system helped record intrinsic or voltage-sensitive dye signals by means of optical imaging in an experiment of more than a year duration, during which the tested monkeys and the implanted substitute were both in superb condition, with no evidence of damage [39].

Combined with modern electronic technologies, artificial dura systems have gradually developed into complex chamber systems that integrate signal measurement and processing. A chamber-based neurotechnology device testing approach is portrayed in Figure 3b. The dura is replaced with one containing embedded ECoG arrays, and the chamber sealing specializes the cable routing, which avoids extra surgical implantation and allows for rapid test cycles with reversible seals, instead of the traditional techniques of repeating the animal preparation, device placement, and data acquisition. Neural activity is successfully observed in the form of evoked potentials by the optimized system when the subject experiences full-field visual stimuli or performs delayed-reach tasks [25]. Figure 3c shows the design of a chamber to house a silicone artificial dura with custom μECoG arrays and a commercially available micro-drive system with penetrating electrodes, where these two parts are precisely aligned. Furthermore, PEEK plastic is used to maintain the imaging compatibility, and the system is affixed to the skull via MetaBond and dental acrylic. It is notable that μECoG, LFP, and spiking activity are simultaneously recorded via one system, and the modular design can incorporate other functional hardware in principle [40].

Figure 3d presents an ideal model of an artificial dura system for long-time implantation, which involves a variety of smart built-in modules, including micro sensor nodes, data processing units, and wireless communication modules [41]. Taking the first step towards this model, a primitive overlaid cranial window based on 3D printing was engineered and implanted into mice for 21 weeks. In this prototype, the Ti honeycomb is coated with polydimethylsiloxane (PDMS), allowing for optical imaging and transcranial injection [42].

### 3.2. Catheter-Based Systems

Catheter-based devices are another type of universally applied interface for medical applications such as endoscopic surgery and catheterization procedures. A conventional catheter-based device in medicine is composed of tubes to give drugs or take samples and sheaths or balloons to provide the mechanical force for fixation or dilation [43]. So far, some progress has been made in enhancing their procedural security and improving the device stability [44,45,46]. But previous systems have been focused on the capability to infuse or drain fluids and have neglected the possibility of realizing signal detection.

**Figure 4:**
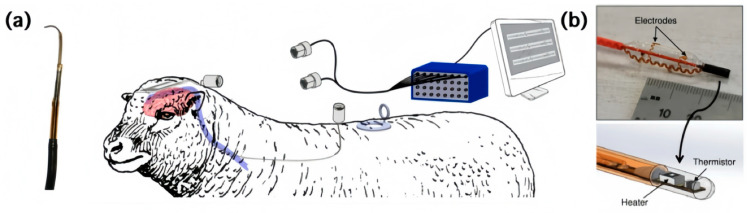
Examples of catheter-based systems. (**a**) Image of a coaxial catheter system including sheaths and microwire and schematic drawing of the animal experiment [47]; (**b**) optical image of a balloon-based catheter system and expanded drawing of the flow sensor on the tip [48].

Recent advances in catheter-based systems have added electrical modules, making them more intelligent and controllable. Figure 4a introduces an endovascular system which can be simply summed up as a passive stent-electrode recording array wrapped in coaxial catheters. The microwire and outside catheters are navigated to the target vessel using the Seldinger technique and digital subtraction angiography, tunneled subcutaneously to a custom-made hermetic connector, which is secured to a muscle, and exiting the skin into a percutaneous micro circular plug. This work reported the recording of vascular electrocorticography in living sheep for up to 190 days [47]. In Figure 4b, the arterial sheaths facilitate the deployment of a balloon-based catheter system into the femoral artery. The system is built on a flexible balloon substrate that gently makes contact with the surrounding tissues when inflated with cold saline. The electrodes and flow sensors positioned on the catheter are connected in wavy geometry using a copper or gold pad between two PI layers. This system offers a way to evaluate vessel health, which was proven by signal acquisition and analysis of the blood flow in anesthetized farm swine [48].

## 4. Further Discussion

### 4.1. Perforation Site

The selection of the perforation site is a critical consideration for the design of interfaces, depending on the requirements of the devices and the conditions of the subjects. Considering the signal attenuation with distance in wired transmission, the interface usually pierces the nearby tissues around the target organ to ensure high-quality interaction between the internal and external devices. This kind of perforation not only simplifies the surgical procedure for implants owing to concentrated incisions but also diminishes the risk of complications, as the implants being in proximity to the target organ avoids other important vulnerable structures such as the nerves and blood vessels. Voluntary actions of the subjects influence the selection of the perforation site as well. In many testing experiments, the interfaces are placed on the head or the back of an animal so that they do not impede small-scale movement nor will be displaced by frequent touching [49]. Faced with the chosen perforation sites, the interfaces utilize tailored designs. For example, an interface on the abdomen is expected to be softer and more stretchable than that on the skull on account of biomechanical stresses.

In summary, the selection of the perforation site involves a comprehensive assessment of signal transmission, surgical feasibility, subject behavior, and anatomical considerations. By carefully considering these factors and employing tailored design approaches, implantable interfaces can achieve the optimal performance and integration with living tissues.

### 4.2. Fixing Strategy

An ignored problem for interfaces crossing living tissues is the technique for fixing the piercing part into the biological tissue. For tests where the experimental animals are continuously anesthetized or tied to a pad, the skin incisions are closed with sutures after subcutaneous implantation, and the tether is thus constrained at the seam [50,51,52,53]. Nevertheless, this method is inapplicable when the test is conducted on conscious unrestrained animals, in which case the interface will easily fall off. Screws are also used, but this method is only suitable for rigid interfaces and perforation sites like the skull [54,55]. A more common solution is to apply dental or bone cement to cover the contact area between the interface and tissue. The cement, on the one hand, possesses high tensile strength, which effectively prevents displacement of the interfaces [56]. On the other hand, it protects the surrounding tissue from invaders and prevents the release of exudate thanks to its good sealing ability. Since the interface has direct contact with the surgical wound, an ideal interfacing strategy is supposed to help reduce inflammation or rejection. Therefore, cement with antibiotics has been employed to ensure the duration of live experiments [57]. Despite all these advantages of cement, it has a fatal flaw: it is relatively hard compared to soft tissues, which may cause acute injury such as tissue displacement and glial activation, leading to both tissue and device damage [58,59].

To address these challenges, novel fixation strategies are being explored. These include the development of softer, more biocompatible materials that can securely anchor the interface while minimizing tissue trauma. Additionally, advancements in bioadhesive technologies aim to provide strong yet flexible adhesion to living tissues without causing undue harm. By developing solutions that balance secure attachment with tissue compatibility, researchers can enhance the safety and effectiveness of implantable bioelectronics.

### 4.3. Long-Term Indwelling

The testing animals are often killed after temporary experiments to further examine their tissues. But when it comes to wired systems for constant monitoring, the implantable devices and the surface interfaces must have prolonged retention in vivo, which may lead to device failure and subject damage. This issue can be resolved by choosing a proper material that is compatible with the tissues to isolate the inner module from bodily fluids [60]. Silicone materials, including PDMS, have been widely used, and other emerging materials such as metal–organic frameworks also demonstrate superior performance in device manufacturing [61].

Furthermore, the stability and connectivity of each module is crucial because any irregularity will probably result in surgery to replace the whole system. Recent advancements offer promising solutions to improve the stability and connectivity of implantable systems. For example, researchers have developed biphasic, nano-dispersed interfaces by thermally evaporating conductive nanoparticles into an elastic substrate. This innovative approach provides a plug-and-play connection between different modules, ensuring robustness and flexibility. Such interfaces have demonstrated reliable performance in detecting subcutaneous compound muscle action potential [62], showcasing their potential to enable long-term retention in living tissues while maintaining connectivity with external devices.

By adopting the novel approaches described above, researchers can mitigate adverse outside influences and enhance the reliability and longevity of implantable devices for continuous monitoring applications. These advancements facilitate safer and more effective long-term studies in both animal models and human subjects.

### 4.4. Wireless Strategy

The interfaces introduced above, however, prompt long-term opening of wounds, increasing the danger of infection. Other complications with wires like thrombosis and organ malfunction are also potential hazards that have received considerable attention [63,64]. In addition, wired connection to external devices restricts the behaviors of the test subjects, especially their social interaction and activity level [65]. By contrast, wireless implantable systems minimize these negative environmental impacts because of the postoperative suture and greatly reduce mechanical constraints so that the subjects are free to move around [66]. As a well-known example, Neuralink displayed a live pig with a coin-sized device inserted into its brain on a webcast on 28 August 2020 [67]. This device could detect neural spikes and send wireless signals to a nearby computer, and the pig could freely eat or sniff on camera. After these early animal experiments, this year, Neuralink claimed to have implanted a brain chip into a human for the first time, but more detailed information about this trial still remains confidential and undisclosed, which has triggered heated controversy about its safety and reliability [68,69]. Although wireless medical systems have raised high expectations and have already been put into practical use, there still exist some unaddressed tasks [70]. For one thing, living tissues pose obstacles to wireless communication, compelling researchers to explore alternative methods for high-efficiency transmission [71,72]. For another, wireless power transfer approaches need further optimization to completely replace implanted batteries, which have a limited lifespan and entail subsequent replacement [73,74].

## 5. A Proposal for Multi-Functional Interfaces

**Figure 5:**
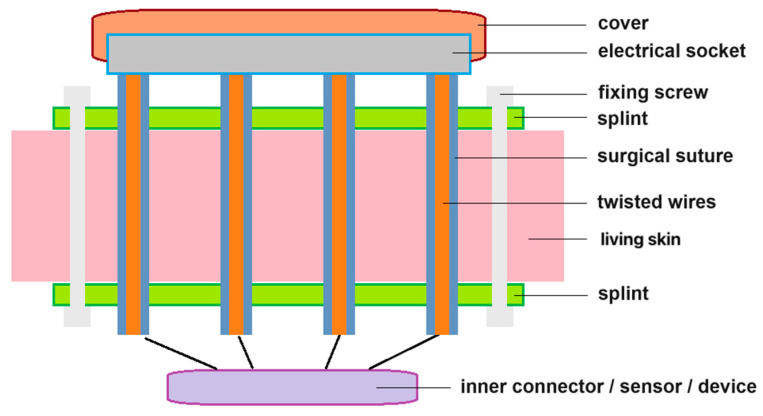
A schematic drawing of a multi-functional transdermal interface.

We present here a primitive approach to a multi-functional electrical interface for living tissues. A cross-sectional image of its central part is shown in Figure 5. The overall structure can be described as two pieces of splint embedded with an array of conductive twisted microwires. The splints are made of biocompatible material to prevent inflammation or rejection of the tissues in direct contact with the interface. The core of each surgical suture is a pair of two twisted conducting microwires (Cu, Au, or Ag), which can also be replaced by an optical micro-fiber. The two splints are fixed together with thin metallic screws (made of Ti or stainless steel). The external devices can incorporate wireless technology by integrating processing modules into the socket casing of this device, further enhancing the function of this device. To prevent wear and tear of the signal channels, a sealing and insulating cover is placed over the interface when the system is in idle status. Applications of this interface are not limited to soft tissues such as the skin. It can also be applied to harder sites such as bones through a resection–implantation approach.

## 6. Conclusions

The above reviews the recent developments in interfacing technologies for implantable bioelectronics. From the structural design to the selection of materials and processes, every aspect has a crucial impact on the efficacy and longevity of the interface. While tethered interfaces offer stability and signal fidelity, non-tethered interfaces such as artificial dura systems and catheter-based systems present innovative solutions for specific applications. However, there are challenges that necessitate concerted and continual efforts.

The selection of the perforation site for interfaces is still in the exploratory stage, aiming to meet the functional requirements of a system while minimizing damage to the organism. We expect that in the future, through the use of imaging techniques and computational modeling, researchers will be able to optimize the decision-making process, mitigating risks to surrounding tissues and structures during the surgical procedure.

The existing fixing strategies at the piercing site also need to be improved upon to achieve non-toxicity to the tissues and reduce infections caused by interactions between internal and external substances. Advances in materials science and bioengineering hold good prospects for developing adhesives and sealants that provide robust fixation while promoting tissue integration and reducing inflammatory responses.

Considering the long-term indwelling of the interfaces, the demand for biocompatible materials, sealing techniques, and connecting methods remains paramount. Novel manufacturing processes, including nanofabrication, offer precise control over the design of the interfaces, ensuring their prolonged retention in vivo.

Moreover, although wireless communication and powering provide assistance to implantable systems, wireless interfaces cannot completely replace wired ones for the time being. Problems such as signal attenuation, tissue interference, and power efficiency continue to pose barriers to seamless integration with implantable devices.

Finally, we present a versatile multi-functional interface model, featuring two micro splints embedded with an array of innovative tethers. It utilizes biocompatible materials to prevent tissue rejection, and its tether design enhances its mechanical strength and facilitates secure communication with external devices.

As the research progresses, interdisciplinary collaboration and technological innovation will be key to overcoming the existing challenges. By leveraging developments in materials science, biotechnology, and electronics, future interfacing technologies may unlock the full potential of implantable bioelectronics. This will pave the way for seamless integration with implantable devices, enabling real-time monitoring of health status, targeted therapy delivery, and improved overall quality of life. By focusing on the development of advanced interfacing technologies, we can overcome the existing challenges and usher in a new era of medical innovation that will benefit patients worldwide.

## Data Availability

The data presented in this study are available on request from the corresponding author.
